# A high-fat diet catalyzes progression to hyperglycemia in mice with selective impairment of insulin action in Glut4-expressing tissues

**DOI:** 10.1016/j.jbc.2021.101431

**Published:** 2021-11-18

**Authors:** Austin M. Reilly, Shijun Yan, Menghao Huang, Surabhi D. Abhyankar, Jason M. Conley, Robert N. Bone, Natalie D. Stull, Daniel J. Horan, Hyun C. Roh, Alexander G. Robling, Aaron C. Ericsson, Xiaocheng C. Dong, Carmella Evans-Molina, Hongxia Ren

**Affiliations:** 1 Stark Neurosciences Research Institute, Medical Neuroscience Graduate Program, Indiana University School of Medicine, Indianapolis, Indiana, USA; 2 Herman B. Wells Center for Pediatric Research, Department of Pediatrics, Indiana University School of Medicine, Indianapolis, Indiana, USA; 3 Center for Diabetes and Metabolic Diseases, Indiana University School of Medicine, Indianapolis, Indiana, USA; 4 Department of Biochemistry & Molecular Biology, Indiana University School of Medicine, Indianapolis, Indiana, USA; 5 Indiana Biosciences Research Institute, Indianapolis, Indiana, USA; 6 Department of Anatomy and Cell Biology, Indiana University School of Medicine, Indianapolis, Indiana, USA; 7 Metagenomics Center, University of Missouri, Columbia, Missouri, USA; 8 Richard L. Roudebush VA Medical Center, Indianapolis, Indiana, USA; 9 Department of Pharmacology & Toxicology, Indiana University School of Medicine, Indianapolis, Indiana, USA

**Keywords:** metabolism, diet, glucose transporter type 4 (GLUT4), insulin resistance, insulin receptor (INSR), diabetes, type 2 diabetes (T2DM), glucose metabolism, DIO, diet-induced obesity, FFPE, formalin-fixed paraffin-embedded, FIRKO, fat-Insr-KO, GIRKO, *GLUT4* promoter-driven insulin receptor knockout, GLUT4, glucose transporter type 4, GSIS, glucose-stimulated insulin secretion, HFD, high-fat diet, Insr, insulin receptor, IPGTT, intraperitoneal glucose tolerance test, LPS, lipopolysaccharide, micro-CT, micro-computed tomography, MIRKO, muscle-Insr-KO, NCD, normal chow diet, OGTT, oral glucose tolerance test, ROI, region of interest, T2D, type 2 diabetes, VLDL, very-low-density lipoprotein

## Abstract

Insulin resistance impairs postprandial glucose uptake through glucose transporter type 4 (GLUT4) and is the primary defect preceding type 2 diabetes. We previously generated an insulin-resistant mouse model with human *GLUT4* promoter-driven insulin receptor knockout (GIRKO) in the muscle, adipose, and neuronal subpopulations. However, the rate of diabetes in GIRKO mice remained low prior to 6 months of age on normal chow diet (NCD), suggesting that additional factors/mechanisms are responsible for adverse metabolic effects driving the ultimate progression of overt diabetes. In this study, we characterized the metabolic phenotypes of the adult GIRKO mice acutely switched to high-fat diet (HFD) feeding in order to identify additional metabolic challenges required for disease progression. Distinct from other diet-induced obesity (DIO) and genetic models (*e.g.*, *db/db* mice), GIRKO mice remained leaner on HFD feeding, but developed other cardinal features of insulin resistance syndrome. GIRKO mice rapidly developed hyperglycemia despite compensatory increases in β-cell mass and hyperinsulinemia. Furthermore, GIRKO mice also had impaired oral glucose tolerance and a limited glucose-lowering benefit from exendin-4, suggesting that the blunted incretin effect contributed to hyperglycemia. Secondly, GIRKO mice manifested severe dyslipidemia while on HFD due to elevated hepatic lipid secretion, serum triglyceride concentration, and lipid droplet accumulation in hepatocytes. Thirdly, GIRKO mice on HFD had increased inflammatory cues in the gut, which were associated with the HFD-induced microbiome alterations and increased serum lipopolysaccharide (LPS). In conclusion, our studies identified important gene/diet interactions contributing to diabetes progression, which might be leveraged to develop more efficacious therapies.

Diabetes imposes substantial healthcare costs and diminished quality of life because of the risk of developing life-threatening diabetic complications (1). The pathophysiology of type 2 diabetes (T2D) is driven by widespread insulin resistance that prevents cellular glucose uptake and causes chronic hyperglycemia (2). The latent period prior to diabetes onset, termed prediabetes, is caused by peripheral insulin resistance and compensatory increases in serum insulin, affecting approximately one in three individuals in the United States in the year 2020 (National Diabetes Statistics Report 2020, https://www.cdc.gov/). The progression to overt diabetes, affecting one in ten individuals, involves β-cell dysfunction and incomplete compensation for insulin resistance. The cascade of events that culminate to overt diabetes is not fully understood, but the undeniable link between obesity and T2D brings into question the role of dietary, genetic, lifestyle, and environmental factors (3).

Glucose transporter 4 (GLUT4)-mediated postprandial glucose uptake influences whole-body glucose homeostasis *via* several tissue-dependent mechanisms. Muscle is responsible for 70–80% of glucose uptake after glucose injection and largely depends on GLUT4 for this function (4, 5). Interestingly, adipose-specific Glut4 deletion decreased the insulin sensitivity of the muscle and liver, resulting in systemic glucose intolerance and insulin resistance (6, 7). Multiple proof-of-principle studies demonstrated the benefits of Glut4 upregulation using overexpression models. Transgenic overexpression of *Glut4* improved glycemia in *db/db* mice (8, 9) as well as during high-fat diet (HFD) challenge (10, 11). Furthermore, overexpression of Glut4 in the muscle or in white adipose conferred benefits to glycemia, insulin sensitivity, and substrate fluxes (7, 12, 13, 14).

Although GLUT4 studies reveal various endocrine and substrate flux pathways related to glucose homeostasis, the phenotypes arising from tissue-specific insulin receptor (Insr)-knockout models were generally milder compared with tissue-specific Glut4-knockout. Muscle-Insr-KO (MIRKO) mice had unexpectedly normal glucose tolerance, insulin sensitivity, and serum concentrations of glucose and insulin (15). Interestingly, Fat-Insr-KO (FIRKO) mice were protected from obesity and glucose intolerance (16). Together, these studies suggest that multiple pathways can stimulate downstream signaling, Glut4 translocation, and cellular glucose uptake, thus revealing a complex landscape of insulin resistance mechanisms that may lead to diabetes.

We previously generated a highly insulin-resistant mouse model with human *GLUT4* promoter-driven insulin receptor knockout (GIRKO) in the muscle, adipose, and neuronal subpopulations. Our previous work shows that Glut4-expressing neurons are abundant in the ventromedial nucleus and arcuate nucleus of the hypothalamus and that Insr signaling in Glut4 neurons regulates peripheral glucose metabolism (17, 18, 19, 20). GIRKO mice exhibit hallmarks of metabolic syndrome arising from insulin resistance: peripheral and central insulin resistance, elevated serum insulin, and elevated hepatic glucose production (17). However, GIRKO mice on normal chow diet (NCD) had a lower-than-expected rate of overt diabetes, which only increased with aging, suggesting that additional factors/mechanisms are responsible for adverse metabolic effects that drive the ultimate progression of overt diabetes.

HFD is associated with body weight/adiposity gain, dyslipidemia, peripheral insulin resistance, and glucose intolerance (21). In the current study, we administered HFD to GIRKO mice to test the hypothesis that GIRKO mice would have accelerated progression of diabetic phenotypes. Distinct from other genetic (*e.g.*, *db/db* and *ob/ob mice*) and diet-induced obesity (DIO) models, GIRKO mice were protected from HFD-induced excessive adiposity gain, which precluded excessive fat mass as a confounding factor. Using GIRKO mice as a model of insulin resistance, we showed that HFD accelerated the progression to diabetic phenotypes and further identified additional factors/mechanisms responsible for adverse metabolic effects that drive the ultimate progression of frank diabetes, including blunted incretin effect, dyslipidemia, and increased gut inflammation.

## Results

### GIRKO mice had lower body weight gain and reduced adiposity throughout high-fat diet (HFD) feeding

**Figure 1:**
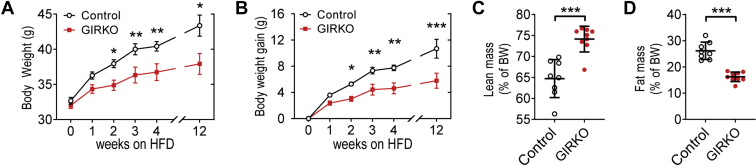
**GIRKO mice had lower body weight gain and reduced adiposity throughout high-fat diet (HFD) feeding.***A*, body weight measured from the beginning of HFD. *B*, change in body weight since beginning HFD. *C*, lean mass percentage of total body weight (BW). *D*, fat mass percentage of total body weight (BW). Data shown are Mean ± SEM. Statistical comparisons in panels *A* and *B* were performed using Fisher's LSD. Student's *t* test was used for panels *C* and *D*. (∗) indicates *p* < 0.05, (∗∗) indicates *p* < 0.01, (∗∗∗) indicates *p* < 0.001, n = 8 Control, 9 GIRKO for all panels.

We assessed total body weight and body composition in adult mice after switching to HFD in order to determine whether GIRKO mice were more susceptible to diet-induced obesity. We measured the body weight change of male Control and GIRKO mice during HFD feeding, which became significantly different after week 2 (Fig. 1, *A* and *B*) and continued to further diverge. After 12 weeks, the body weight gain in male GIRKO mice was roughly half that of Control mice (10.69 ± 1.43 g *versus* 5.79 ± 1.15 g, Fig. 1*B*). Female Control mice had more weight gain and more adiposity than GIRKO mice after longer HFD feeding (Fig. S1, *A–D*). Of note, Control and GIRKO mouse cohorts with matched glycemia and body weight were used for the HFD challenge. EchoMRI scans were used to measure the body composition of the male cohort. On day 25 of HFD feeding, total body weight of GIRKO mice was significantly less (39.67 ± 0.76 g *versus* 35.16 ± 0.59 g). GIRKO mice were leaner overall, having increased percentage of lean mass (Fig. 1*C*) and decreased percentage of fat mass (Fig. 1*D*).

As aging also contributes to adiposity gain, we compared the body composition of age-matched cohorts that were fed either HFD or NCD. Control mice gained weight on HFD (Fig. S2*A*), which mainly was attributed to the adiposity gain (Fig. S2, *B* and *C*). In contrast, GIRKO mice gained less weight on HFD and were leaner (Fig. S2, *D* and *E*).

### GIRKO mice have reduced overall fat mass despite modest differences in adipocyte morphology

**Figure 2:**
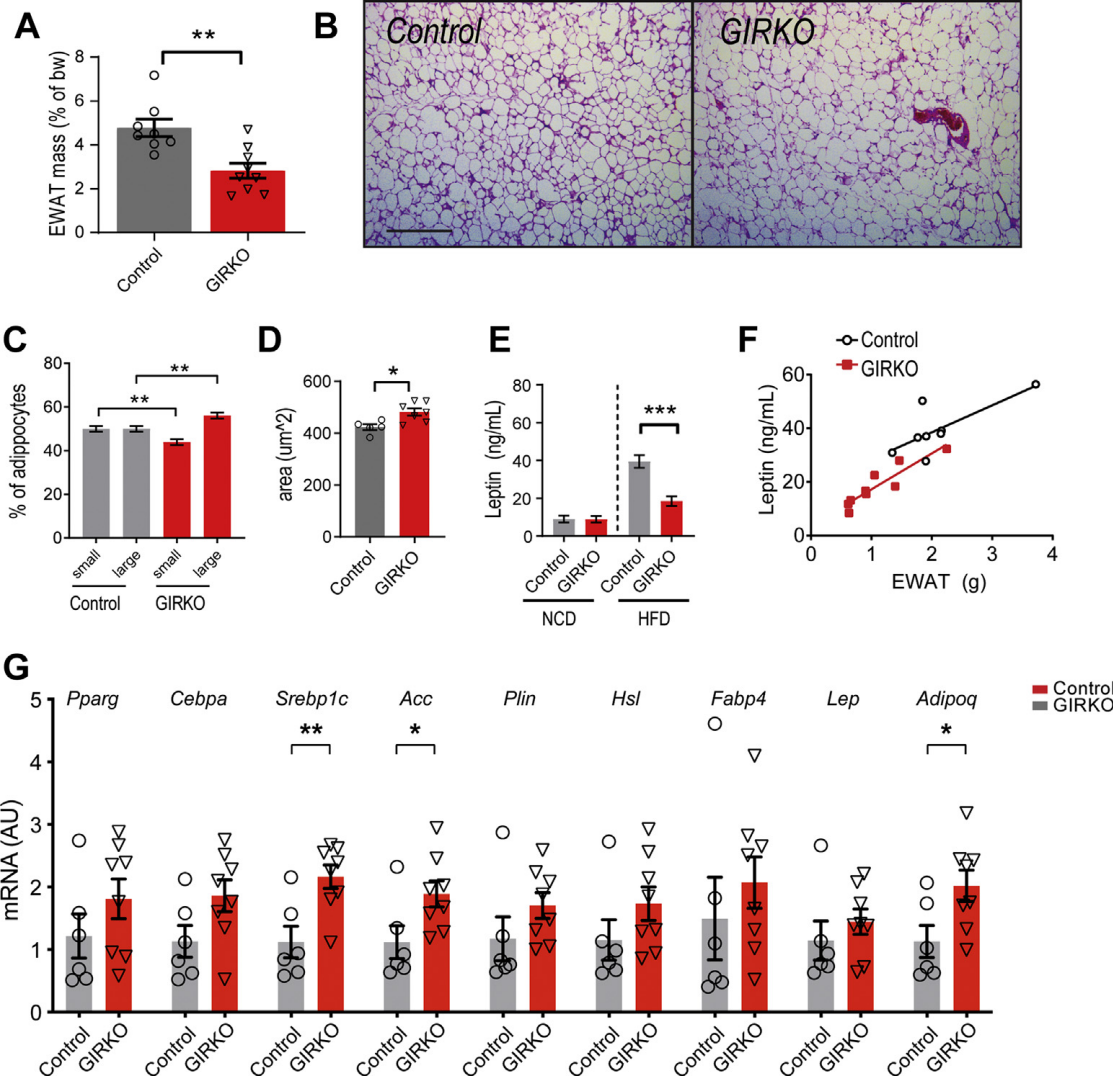
**GIRKO mice have reduced overall fat mass despite modest differences in adipocyte morphology.***A*, epididymal white adipose tissue (EWAT) mass as a percent of body weight (bw) after 14 weeks of HFD exposure. *B*, representative hematoxylin and eosin-stained images of EWAT in control and GIRKO mice. Scale bar is 500 μm. *C*, comparison of the percent of small and large adipocytes in EWAT. The median adipocyte size of Control EWAT was used as a cutoff to determine small or large adipocytes and was set to 324 μm (2). (n = 5 Control, 7 GIRKO). *D*, average area of adipocytes in EWAT. (n = 5 Control, 7 GIRKO). *E*, serum leptin concentration in NCD and HFD cohorts. (n = 8 Control-NCD, 7 GIRKO-NCD, 8 Control-HFD, and 9 GIRKO-HFD). *F*, correlation between serum leptin concentration to EWAT mass. n = 8 Control, 9 GIRKO. *G*, mRNA expression of adipocyte marker genes. Data shown are Mean ± SEM. Statistical comparisons in panels *A*, *D*, and *G* were performed using student's *t* test Statistics in panels *C* and *E* are from two-way ANOVA and Sidak post-hoc test. (∗) indicates *p* < 0.05, (∗∗) indicates *p* < 0.01, (∗∗∗) indicates *p* < 0.001.

Since GIRKO mice were protected from obesity, we measured the epididymal white adipose tissue (EWAT) mass and found that it was decreased (Fig. 2*A*), consistent with reports in adipose-specific Insr-knockout (FIRKO) mice (16). We performed hematoxylin and eosin staining on EWAT tissue sections to characterize the adipocyte morphology (Fig. 2*B*). Comparing to the median adipocyte area in Controls, GIRKO mice had fewer small adipocytes and more large adipocytes (Fig. 2*C*), which increased the average adipocyte size modestly in GIRKO mice (Fig. 2*D*). We measured the gene expression in the adipose tissue of GIRKO and Control mice (Fig. 2*G*). The expression of *Pparg* and *Cebpa*, critical regulators of adipocyte differentiation, was comparable between GIRKO and Control mice. We further examined the expression of genes involved in lipid metabolism. The expression of *Srebp1c*, *Acc*, *Plin1*, and *Hsl* was significantly increased in the GIRKO adipose tissue, while *Fabp4* expression was comparable between GIRKO and Control mice.

Leptin is an adipokine that is normally secreted in proportion to overall fat mass and regulates satiety to maintain body weight homeostasis. Since leptin suppresses appetite, we further sought to determine whether the decrease in body weight was caused by elevated serum leptin. However, we found that serum leptin was decreased in GIRKO mice compared with Control mice and showed a similar correlation between serum leptin and EWAT mass (Fig. 2, *E* and *F*).

### GIRKO mice have aberrant energy partitioning after acutely switching to high-fat diet

**Figure 3:**
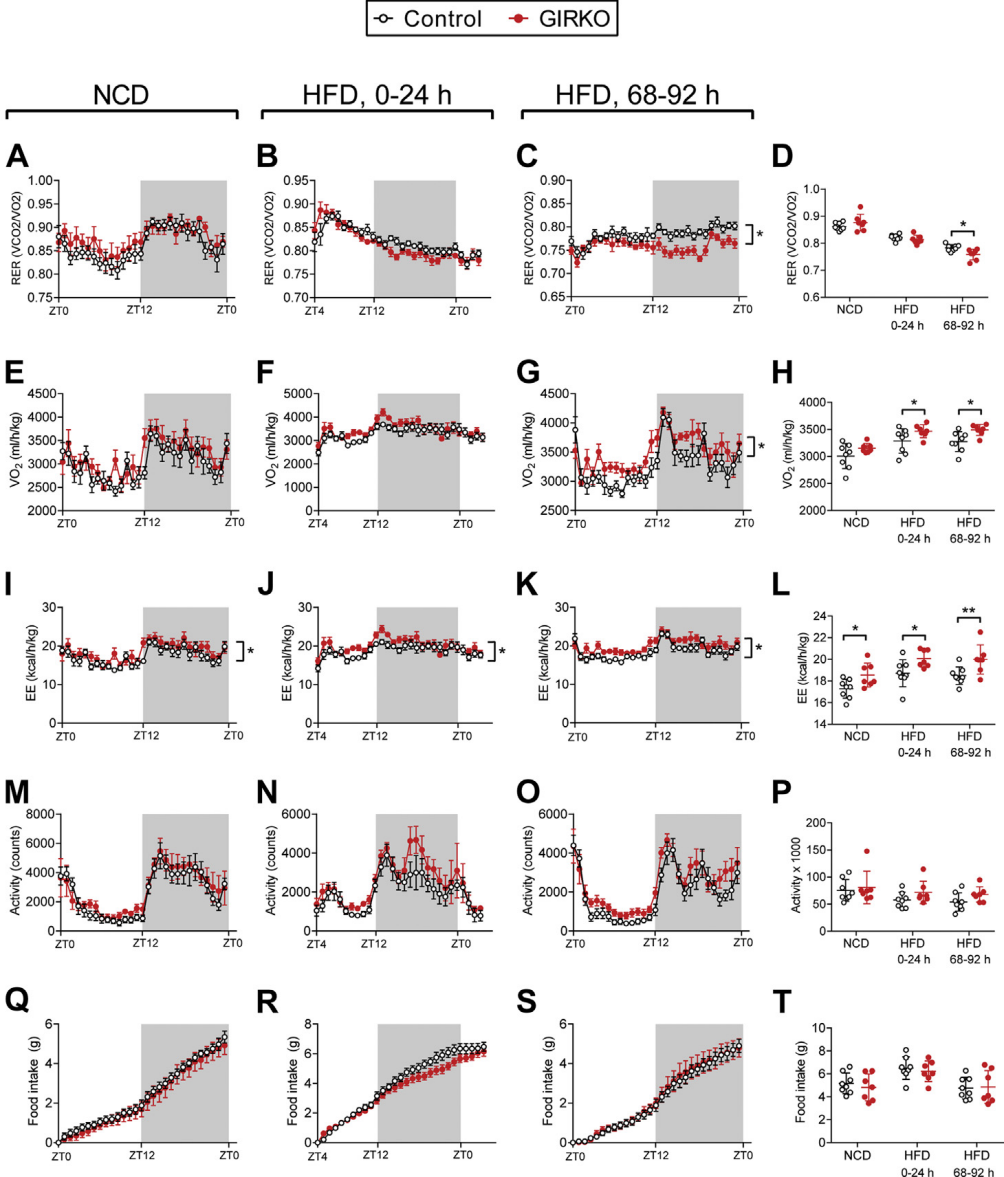
**GIRKO mice have aberrant energy partitioning after acutely switching to high-fat diet (HFD).***A–T*, indirect calorimetry measurements taken before and during HFD administration; 24-h representative recordings are shown for each parameter prior to HFD, for 0–24 h of HFD exposure, and after 68–92 h of HFD exposure. *A–C*, respiratory exchange ratio (RER) measurements. *D*, average RER for each 24-h observation period. *E–G*, oxygen consumption (VO2) measurements. *H*, average VO_2_ for each 24-h observation period. *I–K*, energy expenditure (EE) measurements, normalized to lean body mass. *L*, average EE for each 24-h observation period. *M–O*, horizontal locomotor activity (Act) measured by number of light beam breaks. *P*, total Act counts measured for each 24-h observation period. *Q–S*, cumulative food intake during indirect calorimetry measurements. *T*, total food intake for each 24-h observation period. Data shown are Mean ± SEM. Statistical comparisons in *panels D*, *H*, *L*, *P*, *T* were performed using Fisher's LSD. All other panels were analyzed using two-way repeated measures ANOVA to compare genotypes (detailed statistical information is provided in supporting information). n = 8 Control, 7 GIRKO for all panels. (∗) indicates *p* < 0.05; (∗∗) indicates *p* < 0.01.

In order to determine whether GIRKO mice had altered energy balance, nutrient partitioning, or feeding behaviors, which could otherwise explain decreased weight gain on HFD, we characterized their metabolic features using metabolic phenotyping cages equipped for indirect calorimetry assessments. Prior to HFD exposure, metabolic parameters were similar to control mice (Fig. 3, *left panels*: *A*, *E*, *I*, and *M*), and remained similar for the first 24-h period of HFD feeding (Figure 3, second column: *B*, *F*, *J*, and *N*). Differences in the respiratory exchange ratio (RER) emerged after 72 h of HFD feeding, indicating that GIRKO mice had increased lipid utilization (Fig. 3*D*). GIRKO mice had relatively increased oxygen consumption during HFD treatment, but not with normal chow (Fig. 3, *E*–*H*). GIRKO mice showed increased average 24-h energy expenditure for normal chow, HFD 0–24 h, and HFD 68–96 h (Fig. 3, *I*–*L*). However, locomotor activity was unchanged (Fig. 3, *M–P*). Lastly, for each day measured, GIRKO mice had similar food intake (Fig. 3, *Q*–*T*), suggesting that GIRKO mice were not suppressing food intake in order to maintain constant body weight.

Taken together, these indirect calorimetry data suggest that GIRKO mice develop perturbations in energy partitioning while consuming HFD, including increased energy expenditure, oxygen consumption, and increased lipid oxidation. The changes to energy balance were relatively modest overall. Therefore, the reduced adiposity of GIRKO mice on HFD was likely a direct result of impaired anabolic function of insulin in the adipose tissue.

### GIRKO mice develop severe insulin resistance and hyperinsulinemia on high-fat diet

**Figure 4:**
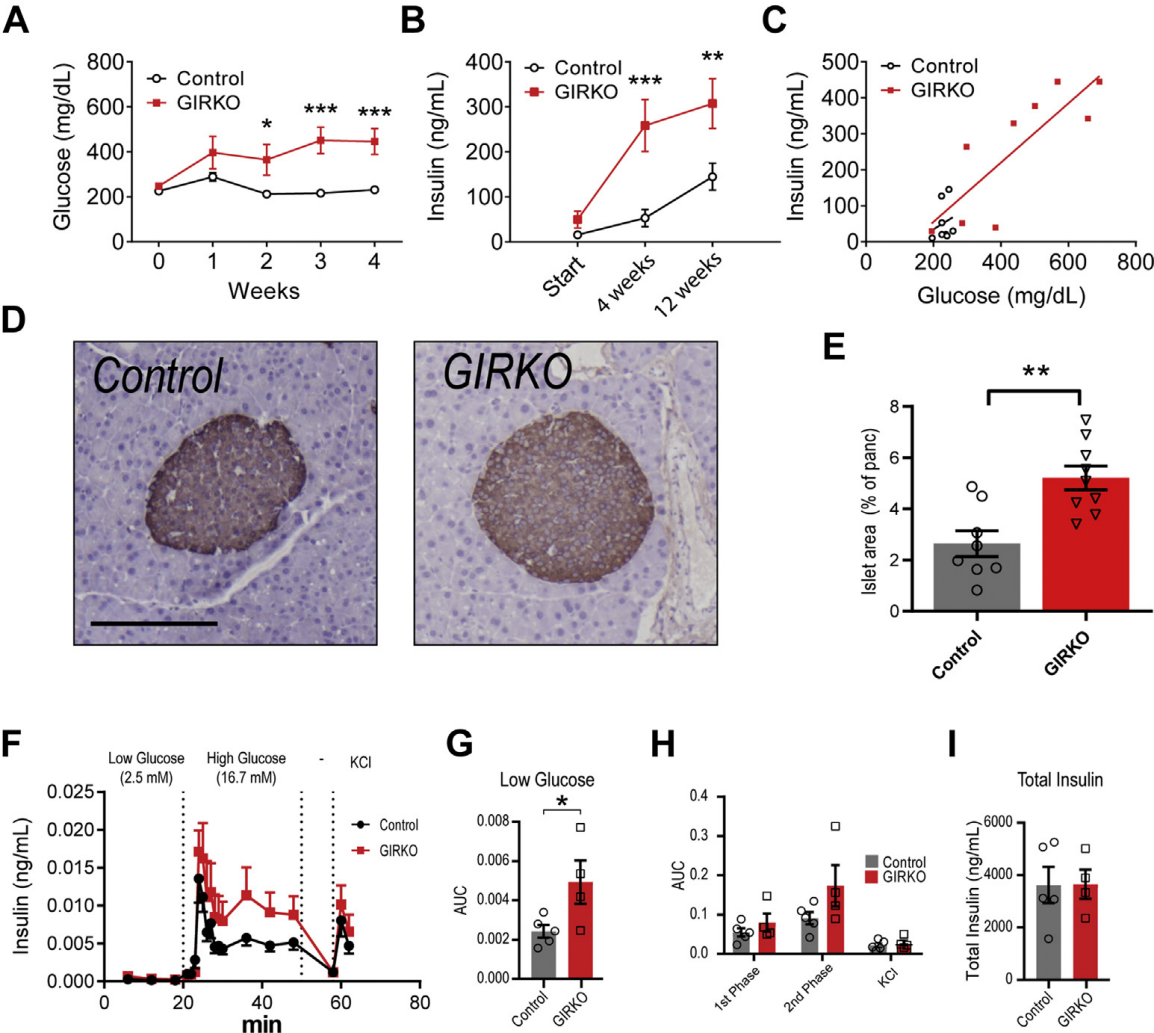
**GIRKO mice develop severe insulin resistance and hyperinsulinemia on high-fat diet (HFD).***A*, *ad libitum* blood glucose measured weekly after starting HFD feeding. *B*, *ad libitum* insulin measured after starting HFD feeding. *C*, *ad libitum* insulin *versus* blood glucose in mice given HFD for 4 weeks. *D*, representative images of insulin staining in pancreatic tissue sections in Control and GIRKO mice. Scale bar is 200 μm. *E*, insulin-positive islet area, as a percent of the total area of the pancreatic section after 14 weeks of HFD. *F–I*, glucose-stimulated insulin secretion (GSIS) in an *ex vivo* islet perifusion system. *F*, insulin concentration measured in perifusates collected at varying concentrations of glucose. Following washout (−), islet cells were depolarized with potassium chloride (KCl). *G*, the area under the curve (AUC) of low glucose perifusion phase. *H*, AUC of first phase (20–30 min), second phase (30–50 min), and KCl (58–62 min) time points. *I*, total insulin concentration in islet lysates collected after perifusion. Data shown are Mean ± SEM. Statistical comparisons in panels *A* and *B* were performed using Fisher's LSD; student's *t* test was used for *panel E*. (∗) indicates *p* < 0.05, (∗∗) indicates *p* < 0.01. n = 8 Control, 9 GIRKO for panels *A–E*. n = 5 Control, 4 GIRKO for panels *F–H*.

We monitored blood glucose and insulin throughout the HFD feeding regimen to determine whether HFD exacerbated insulin resistance in GIRKO mice. GIRKO mice had a trend for increased *ad libitum* blood glucose and insulin on day 0, which did not reach statistical significance (Fig. 4, *A* and *B*). However, after starting HFD, blood glucose was elevated in GIRKO mice starting at week 2 that continued to diverge for weeks 3 and 4 (Fig. 4*A*). After 4 weeks of HFD, serum insulin in GIRKO mice was five times the level of control mice (53.0 ± 18.9 ng/ml *versus* 258.5 ± 57.6 ng/ml, Fig. 4*B*). A plot of insulin *versus* blood glucose after 4 weeks of HFD feeding shows that most GIRKO mice were unable to reduce blood glucose *via* increased insulin secretion (Fig. 4*C*). Immunohistochemical staining of insulin in the pancreas revealed islet hyperplasia in GIRKO mice, suggesting that GIRKO mice increased β-cell mass to produce more insulin and compensate for reduced insulin action in the periphery (Figs. 4, *D* and *E* and S5).

In order to determine whether GIRKO mice had impaired islet function, we performed *ex vivo* islet perifusion experiments that tested the glucose-stimulated insulin secretion (Fig. 4*F*). GIRKO islets in the low glucose media had greater insulin secretion, indicating higher baseline insulin secretion (Fig. 4*G*). The first phase of glucose-stimulated insulin secretion (GSIS) in Control and GIRKO islets was similar (Fig. 4*H*), while the second phase had a trend for increased GSIS that did not reach statistical significance (Fig. 4*H*). Following membrane depolarization with KCl, there was no difference in KCl phase GSIS (Fig. 4*H*). The area under the curves for first-and second-phase GSIS were similar between groups (Fig. 4*H*), and the total insulin content in islet lysates was similar between GIRKO and Control islets (Fig. 4*I*).

### GIRKO mice fed high-fat diet develop dyslipidemia and impaired hepatic glucose metabolism

**Figure 5:**
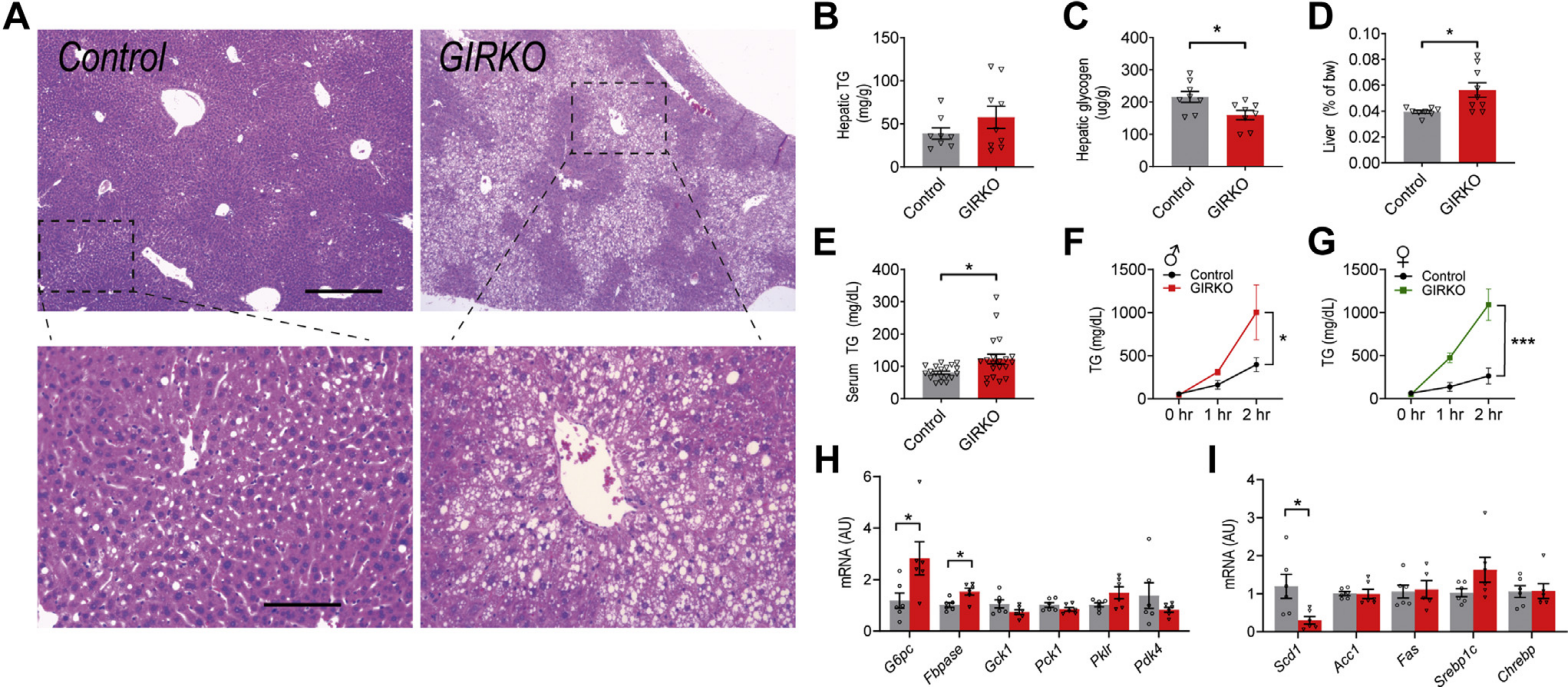
**GIRKO mice fed high-fat diet (HFD) develop dyslipidemia and impaired hepatic glucose metabolism.***A*, representative hematoxylin and eosin stain of hepatic tissue in Control and GIRKO mice. Zoomed-in images are shown in *bottom panels*. Scale bar for *panel A*, top: 500 μm, *bottom*: 125 μm. *B*, hepatic triglyceride (TG) mass per gram of liver (n = 8 Control, 9 GIRKO). *C*, hepatic glycogen mass per gram of liver (n = 8 Control, 9 GIRKO). *D*, liver mass as a percent of body weight (bw) after 14 weeks of HFD feeding (n = 8 Control, 9 GIRKO). *E*, serum triglyceride (TG) concentration during ad libitum HFD feeding (n = 19–20 per group). *F*, hepatic VLDL secretion assay in male HFD cohorts (n = 5 Control, 3 GIRKO). *G*, hepatic VLDL secretion assay in female HFD cohorts (n = 7 Control, 7 GIRKO). *H*, hepatic expression of genes involved in glucose metabolism and homeostasis (n = 6 Control, 6 GIRKO). *I*, hepatic mRNA expression of genes involved in lipid metabolism and *de novo* lipogenesis (n = 6 Control, 6 GIRKO). Data shown are Mean ± SEM. Statistical comparisons in panels *B–D*, *H*, *I* were performed using student's *t* test. In *panels F* and *G*, two-way repeated measures ANOVA was used for genotype comparisons. (∗) indicates *p* < 0.05, (∗∗∗) indicates *p* < 0.001.

We used hematoxylin and eosin staining to determine whether GIRKO mice developed more severe liver steatosis (hepatocyte ballooning and accumulation of lipid droplets) compared with Control mice. Indeed, we discovered that only one out of seven Control mice fed HFD had detectable steatosis, while five out of eight GIRKO mice had varying degrees of steatosis (Figs. 5*A* and S3). Hepatic triglyceride levels trended up in GIRKO mice, though not significantly different from Control mice (Fig. 5*B*). Consistent with the biochemical measurement, we observed a trend of increased Oil Red O staining in the GIRKO hepatic tissue sections (Fig. S4). Hepatic glycogen was less in GIRKO mice (Fig. 5*C*). GIRKO liver weight was significantly higher than that of control mice on HFD (Fig. 5*D*). Together, these results suggest that GIRKO mice had impaired lipid homeostasis and that liver enlargement was caused by increased hepatic lipid content.

We further investigated this and found that GIRKO mice had significantly higher blood triglyceride on HFD (Fig. 5*E*), indicating increased hepatic lipid secretion. Indeed, both male and female GIRKO mice on HFD had significantly higher hepatic very-low-density lipoprotein (VLDL) secretion than their Control counterparts (Fig. 5, *F* and *G*). Of note, female Control and GIRKO mice had comparable body weight at the time of assay, suggesting that the different VLDL secretion result was not due to body weight differences**.** In addition, we measured the expression of hepatic genes involved in the regulation of glucose and lipid metabolism. *G6pc* and *Fbpase* encode rate-limiting enzymes for hepatic gluconeogenesis; both were increased in GIRKO mice, and may have contributed to hyperglycemia (Fig. 5*H*). We observed no change of gene expression in the *de novo* lipogenesis pathway (Fig. 5*I*).

### Glucose intolerance in GIRKO mice is exacerbated by high-fat diet

**Figure 6:**
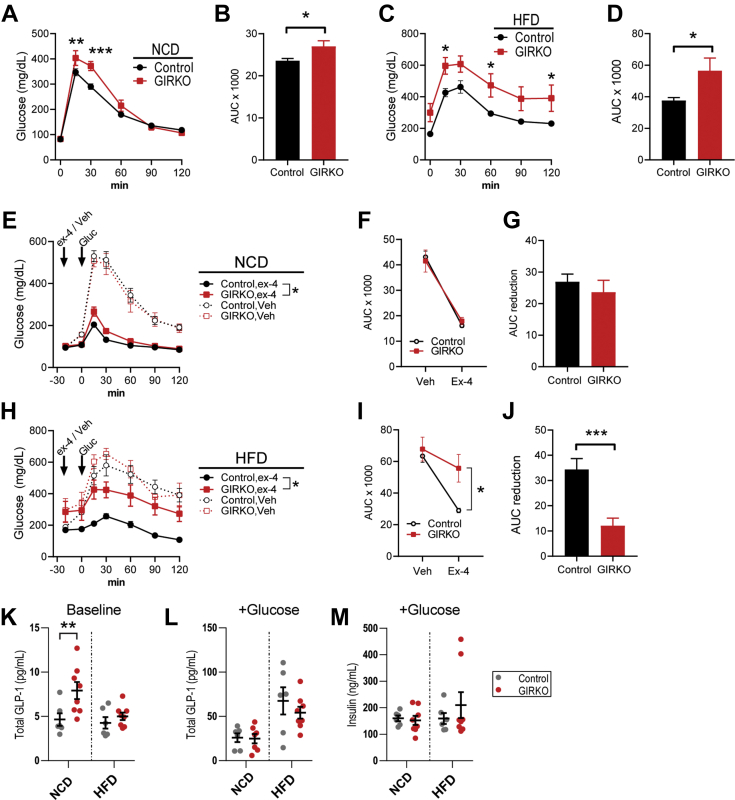
**Glucose intolerance in male GIRKO mice is exacerbated by high-fat diet (HFD).***A*, oral glucose tolerance test (OGTT, 2 g/kg) in normal chow diet (NCD)-fed mice. *B*, area under the curve (AUC) of *panel A*. *C*, oral glucose tolerance test (OGTT, 2 g/kg) in mice given HFD for 13 weeks. *D*, area-under-the-curve (AUC) of *panel C*. *E*, in NCD-fed mice, intraperitoneal glucose tolerance test (IPGTT, 3.3 g per kg lean body mass). The same mice were given pretreatment with vehicle (veh) or exendin-4 (ex-4) on different days. *F*, AUC for *panel E*. *G*, AUC reduction for *panel E*, determined by the difference between vehicle and exendin-4 AUC. *H*, in HFD-fed mice, intraperitoneal glucose tolerance test (IPGTT, 3.3 g per kg lean body mass). The same mice were given pretreatment with vehicle (veh) or exendin-4 (ex-4) on different days. *I*, AUC for *panel F*. *J*, AUC reduction for *panel F*, determined by the difference between vehicle and exendin-4 AUC. *K* and *L*, serum GLP-1 concentration immediately before (*K*) and after (*L*) oral glucose gavage. *M*, serum insulin concentration 10 min after oral glucose gavage. Data shown are Mean ± SEM. Statistical comparisons in panels *B*, *D*, and *J* were performed using student's *t* test. Fisher's LSD was used in panels *A*, *C*, and *I*. For panels *E* and *H*, exendin-4 treated groups were compared using two-way ANOVA. (∗) indicates *p* < 0.05 (∗∗) indicates *p* < 0.01, (∗∗∗) indicates *p* < 0.001. n is indicated in each panel.

In order to evaluate insulin action and peripheral glucose uptake of GIRKO mice, we performed glucose tolerance tests in mice before and after HFD feeding. Oral glucose tolerance was impaired in male NCD-fed GIRKO mice (Fig. 6, *A* and *B*). Oral glucose intolerance was exacerbated by HFD feeding, as evidenced by elevated glucose excursion and increased area under the curve (Fig. 6, *C* and *D*). Female HFD-fed GIRKO mice also showed impaired oral glucose tolerance (Fig. S1*E*). Based on impaired oral glucose tolerance, we hypothesized that the incretin effect of GIRKO mice was blunted. However, we could not rule out the possibility that gut glucose absorption was altered. Therefore, we performed glucose tolerance tests in conjunction with exendin-4 pretreatment in order to observe the glucose-lowering effects of exogenously administered incretin in GIRKO mice. Indeed, the glucose-lowering effect of exendin-4 was severely blunted, especially in GIRKO mice fed HFD (Fig. 6, *E*–*J*). We measured endogenous serum GLP-1 in fasting mice, observing a significant increase in NCD-fed GIRKO mice (Fig. 6*K*). After oral glucose gavage, serum GLP-1 was similar (Fig. 6*L*), suggesting that GIRKO mice had proper glucose-stimulated GLP-1 secretion. We found that oral glucose gavage did not evoke greater insulin secretion in GIRKO mice on either NCD or HFD (Fig. 6*M*). Taken together, we concluded that GIRKO mice have specific defects related to oral glucose tolerance that are partly caused by blunted incretin effects.

### Bone morphometry analysis in GIRKO mice

Diabetes is associated with decreased bone mineralization and poor biomechanical strength (22). Glut4 facilitates insulin-stimulated glucose uptake in osteoblasts; however, ablating Glut4 in the bone did not affect bone morphology or function (23). Considering the importance of bone health in the context of diabetes, we used micro-CT scans to analyze the bone morphometry in GIRKO mice. After 14 weeks of HFD, GIRKO mice had similar bone architecture to control mice (Table S1). In the distal femur, trabecular strut number (Tb.N) was significantly greater and trabecular separation (Tb.Sp) was significantly reduced in GIRKO mice compared with controls. Those indices suggest slightly improved cancellous properties among GIRKO mice. However, the other trabecular parameters (BV/TV, Tb.Th, BMC) and the cortical parameters (BMD, Cort.Th, bending and torsional moments, bone tissue areas) failed to reach statistical significance when comparing GIRKO to controls. Taken together the femoral structural data suggest little to no difference in skeletal properties between GIRKO and Control mice.

### Gut microbiome composition is mainly determined by dietary intervention in GIRKO mice

The gut microbiome, which is composed of microbial communities in the gastrointestinal (GI) tract, regulates the metabolism of the host through several mechanisms and is also a source of lipopolysaccharide (LPS). Increased serum LPS can contribute to inflammatory pathways that inhibit the insulin signaling cascade (24, 25, 26, 27). Additionally, gut bacteria digest complex polysaccharides and fiber to produce short-chain fatty acid (SCFA) by-products that are signaling and nutrient molecules for maintaining the host metabolic homeostasis (28, 29).

**Figure 7:**
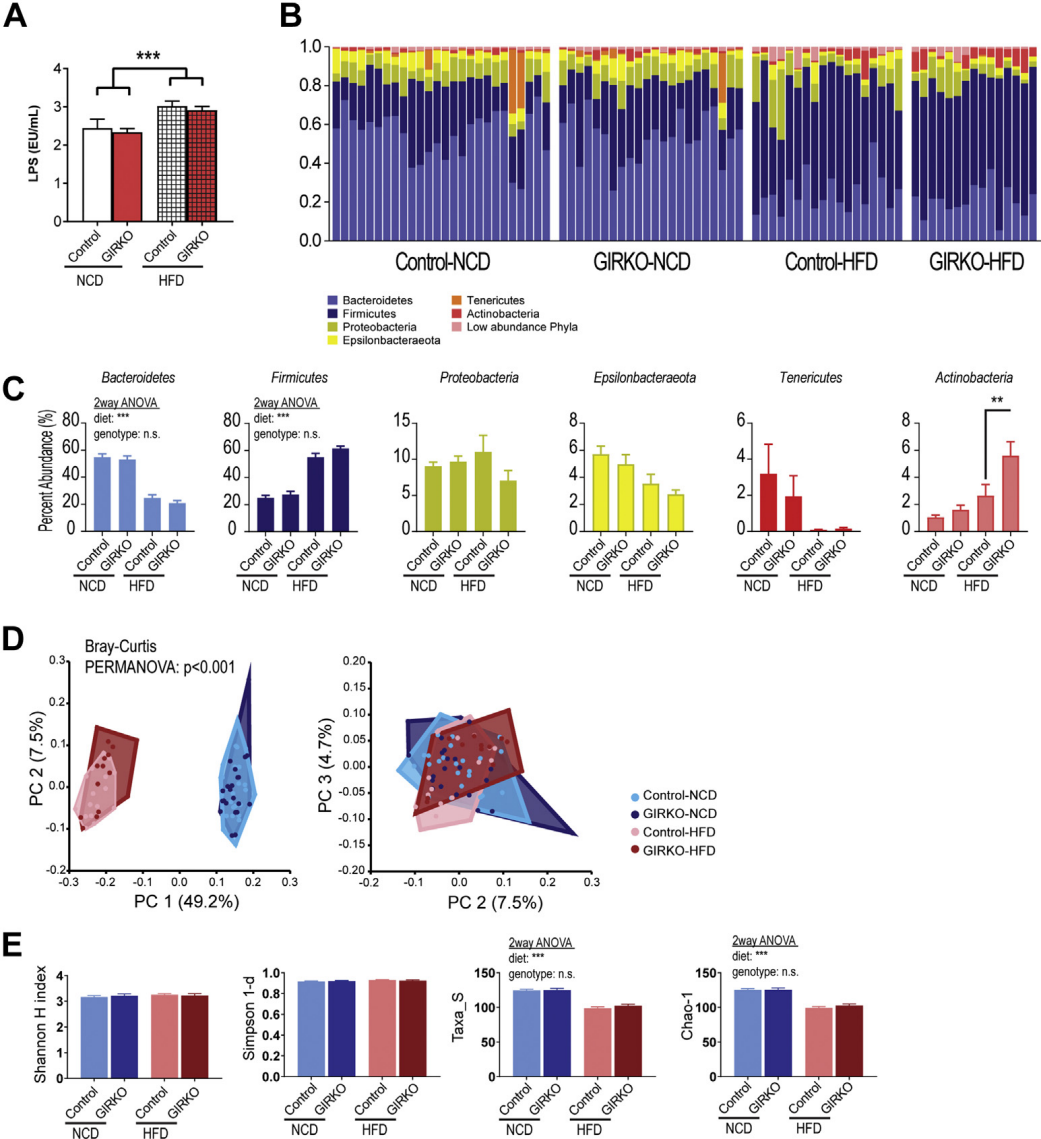
**Gut microbiome composition is mainly determined by dietary intervention in GIRKO mice.***A*, lipopolysaccharide concentration in serum (from *left* to *right*, n = 6, 8, 6, 8). *B*, the relative abundance of each phylum of gut microbes in Control and GIRKO mice fed normal chow diet (NCD) or high-fat diet (HFD). Phyla with percent abundance lower than 1% were grouped as low abundance phyla. *Top*, stacked bar charts, each bar representing an individual mouse sample. *Bottom*, pie charts showing the averaged relative abundances per phyla for each experimental group. *C*, average relative abundance per phyla for each experimental group. *D*, Bray–Curtis principal coordinates (PC) analysis of the microbiome compositions. *Top*, Plot of PC 1 *versus* 2. Bottom: Plot of PC 2 *versus* PC 3. *E*, indices of microbiome alpha-diversity and richness. From *left* to *right*: Shannon index (alpha-diversity), Simpson 1-d (alpha-diversity), Taxa_S (alpha-diversity), Chao-1 index (richness). Bar graphs are Mean ± SEM. In *panel C*, statistics were performed using two-way ANOVA and Tukey's post-hoc test between genotypes on the same diet. (n.s.) indicates not significant, (∗∗) indicates *p* < 0.01, (∗∗∗) indicates *p* < 0.001. n = 26, 22, 18, 15, respectively for Control-NCD, GIRKO-NCD, Control-HFD, and GIRKO-HFD.

We measured the serum LPS concentration in our mouse cohorts and found that, in agreement with existing literature, HFD treatment increased the concentration of LPS in the serum and to a similar level in both Control and GIRKO mice (Fig. 7*A*). This result suggests that our HFD cohorts recapitulated the increase of HFD-induced gut permeability (“leaky gut”) allowing LPS from the microbiota to enter circulation (30, 31).

In order to measure whether intestinal inflammation was altered in GIRKO mice, we performed RNA sequencing (RNA-seq) and gene ontology analysis using intestinal tissues (Table 1). We found that top pathways included “LPS/IL-1 Mediated Inhibition of RXR function” and “Altered T cell and B cell signaling in Rheumatoid Arthritis,” suggesting that inflammatory pathways were altered in GIRKO mice on HFD. Interestingly, “Xenobiotic Metabolism PXR Signaling Pathway” was also identified as a top pathway, suggesting the involvement of gut microbes, which generate xenobiotic molecules. Lastly, lipid metabolism and molecular transport were significantly affected, likely resulting in altered absorption of nutrients in the gut to compensate for the altered pathophysiological state of GIRKO mice on HFD.

**Table 1:** GIRKO mice have increased expression of inflammatory pathway genes in the duodenum

Top canonical pathways	−log(*p*)	z score	Overlap	Molecules
LPS/IL-1 Mediated Inhibition of RXR Function	6.04	1.89	16/225	Abcc2, Acsl6, Aldh4a1, Ces2, Chst3, Cyp2b6, Cyp2c8, Cyp2c9, Fabp5, Gsta3, Gstm3, Il1rl1, Il1rn, Il33, Maoa, Tlr4
Altered T Cell and B Cell Signaling in Rheumatoid Arthritis	4.81	-	9/90	Csf2, Cxcl13, Hla-A, Il1rn, Il33, Spp1, Tlr2, Tlr4, Tnfsf11
Xenobiotic Metabolism PXR Signaling Pathway	4.79	−1.39	13/192	Abcc2, Aldh4a1, Ces1g, Ces2, Chst3, Cyp2b6, Cyp2c8, Cyp2c9, Gsta3, Gstm3, Maoa, Ppp1r3d, Ugt8
Nicotine Degradation III	3.5	−1.63	6/57	Adh7, Csgalnact1, Cyp2b6, Cyp2c18, Cyp2c8, Cyp2c9
Estrogen Biosynthesis	3.25	−2.24	5/42	Cyp2b6, Cyp2c18, Cyp2c8, Cyp2c9, Hsd17b13

Top Upstream regulators	−log(p)	Regulation
Beta-estradiol	11.6	Activated
AGT	10.0	Activated
IL6	9.4	Activated
Dexamethasone	8.9	-
IL10RA	8.6	-

Molecular and cellular functions	*p* value	# Molecules
Lipid Metabolism	1.07E-03–5.77E-10	79
Small Molecule Biochemistry	1.35E-03–5.77E-10	114
Molecular Transport	1.28E-03–1.67E-08	114
Cellular Movement	1.42E-03–1.26E-07	106
Cellular Development	1.42E-03–2.18E-07	89

Differential gene expression between GIRKO-HFD and Control-HFD mice was analyzed in Ingenuity Pathway Analysis (Qiagen) to identify “Top Networks,” “Top Upstream Regulators,” and “Molecular and Cellular Functions.”

Then, we performed 16S rRNA sequencing of fecal bacteria to survey the composition of the gut microbiomes. To determine the dietary effects in GIRKO mice, we collected fecal samples before and after 4 weeks of HFD. Age-matched NCD cohorts were also included. In 81 fecal samples from control and GIRKO mice on NCD and HFD, we observed 1942 amplicon sequence variants (ASVs) among 7.8 million reads. In order to control for the different number of reads between samples, we normalized data to 53,576 reads per sample (4.3 million reads total) and 321 ASVs. We discovered that the relative abundance of microbes was changed by HFD (Fig. 7*B*) and statistically significant for several phyla. The relative abundance of *Actinobacteria* was significantly different (Tukey post-hoc analysis) between GIRKO mice and Control groups fed HFD. However, most bacterial phyla did not differ in relative abundance between genotypes, including: *Bacteroidetes*, *Firmicutes*, *Proteobacteria*, *Epsilonbacteraeota*, *Tenericutes* (Fig. 7*C*). We performed principal coordinate analysis (PCoA) based on the Bray–Curtis distances to identify differences in beta-diversity between treatment groups. We determined using PERMANOVA followed by pairwise statistical tests that the HFD-fed mice had different microbiome compositions compared with the NCD-fed mice, but that no genotype-specific differences were identified for mice on the same diet (Fig. 7*D*). Furthermore, we determined the alpha diversity and richness in each sample. We observed that Shannon H and Simpson-1d indices were not different between treatment groups, suggesting that the alpha-diversities were similar (Fig. 7*E*). However, Taxa-S (observed richness) and Chao1 indices (predicted true richness) were both decreased in HFD groups, which indicate a reduction of the richness of the microbiome (Fig. 7*E*). Taken together, we concluded that differences in the microbiome compositions were mainly driven by diet, with the exception of a significant increase in the abundance of *Actinobacteria* in HFD-fed GIRKO mice.

## Discussion

Despite the established role of insulin in glycemia regulation and the pathophysiology of type 2 diabetes, tissue-specific Insr knockout models often depict certain important aspects of diabetic syndrome. Several insulin-sensitive tissues regulate glucose homeostasis, and compensation for insulin resistance can occur through multiple mechanisms. Additionally, Insr-KO models may require second hits to trigger the onset of diabetes. The goal of our study was to investigate whether GIRKO mice, which have *GLUT4-Cre*-driven Insr ablation, would have accelerated progression to diabetes during a dietary challenge consisting of *ad libitum* HFD feeding.

Our previous studies reported the construction of GIRKO mice and the metabolic phenotype of GIRKO mice on NCD. Major tissues involved in hyperglycemia, obesity, and dyslipidemia were examined, including the brain, muscle, fat, liver, and pancreatic islets. Insulin receptor expression was reduced in the brain, especially hypothalamus and hippocampus. Insulin-mediated Akt signaling was impaired in the muscle, fat, and liver. The previous study also qualitatively examined the histology of the liver, adipose and pancreatic islets. GIRKO mice on NCD went on to develop hyperinsulinemia and elevated hepatic glucose production (17), albeit they developed diabetes at a much lower rate than expected. Our studies also established the role of Insr in Glut4 neurons to regulate counterregulatory responses and sensing nutrient and hormonal cues (18, 19). The current study further examined major tissues involved in metabolic regulation and the metabolic phenotypes of GIRKO mice on HFD. Of note, GIRKO developed diabetes at a much higher rate on HFD than those on NCD. Moreover, we examined the potential contribution of GI tract to the pathophysiologic progression of diabetic phenotype in GIRKO mice on HFD, which was not available in previous studies. We report here that the GIRKO mice on HFD exhibited more severe glucose intolerance during oral glucose tolerance test (OGTT), while previous studies reported the glucose tolerance with IPGTT. The impaired oral glucose tolerance was likely a result of impaired incretin (*e.g.*, GLP-1) signaling shown by the poor exendin-4 response. We analyzed the gene expression profile of the intestine from GIRKO mice and revealed increased expression in inflammatory pathways. We also examined the gut microbiome and circulating LPS in control and GIRKO mice on NCD and HFD. Moreover, we further examined other tissues involved in glucose and lipid metabolism. We quantitatively measured EWAT mass, adipocyte cell size, and the expression of critical genes involved in metabolic regulation in the adipose tissue. We quantitatively measured the pancreatic islet size by insulin staining and insulin secretion in perifusion experiment using isolated islets. We quantitatively measured liver lipid and glycogen content, metabolic gene expression, and liver VLDL secretion. Thus, the current study is a significant advancement that illustrates the dietary effect and highlights the contribution of critical tissues in the context of HFD during type 2 diabetes pathological progression.

HFD-fed mice recapitulate several aspects of metabolic syndrome (32, 33) including obesity and insulin resistance. However, there are several examples showing that HFD in the absence of obesity is insufficient to produce insulin resistance. Recently, it was shown that HFD feeding did not produce insulin resistance when weight gain was controlled (34, 35). Similarly, time-restricted feeding of HFD attenuated insulin resistance despite similar food intake (36). Genetic background also contributes to the severity of HFD phenotypes in mice (21, 37).

Although these studies called in to question whether HFD in the absence of obesity can cause insulin resistance, our study definitively shows the impact of underlying insulin resistance in Glut4-expressing cells and how HFD propels disease progression. A potential explanation for diabetes in HFD-fed GIRKO mice, but not other lean models on HFD, is that the plasticity of white adipose tissue may be impaired. Cariou *et al*. (38) found that in order for MIRKO mice to adapt to muscle insulin resistance, white adipose was sensitized to insulin, leading to greater white adipose tissue expansion and greater glucose utilization in adipocytes. Since GIRKO mice lacked Insr in both muscle and adipose, white adipose tissue plasticity was presumably limited.

An important finding in our current study was that GIRKO mice were protected from diet-induced obesity but were not protected from other diet-induced maladies. On NCD, GIRKO mice have diabetic phenotypes, which include (i) random-feeding hyperglycemia, (ii) modest glucose intolerance, (iii) hyperinsulinemia, and (iv) β-cell hyperplasia (17). Remarkably, HFD initiated rapid progression to overt diabetes in nearly all mice, which was after just 2 weeks and in the absence of obesity. The earliest observed metabolic changes were captured using indirect calorimetry at 3 days of HFD exposure. We found that GIRKO mice had similar food intake but had altered energy partitioning for utilizing lipids as fuel, along with modestly increased energy expenditure.

We observed multiple defects to glycemia regulation in GIRKO mice during HFD feeding. First, we observed hyperinsulinemia and hyperglycemia during *ad libitum* feeding. Secondly, GIRKO mice had impaired oral glucose tolerance and exendin-4 glucose-lowering effects. In humans, incomplete compensation for insulin resistance causes β-cell stress and ultimate failure. Blunted incretin effects have been reported in diabetic patients, as measured by the percent increase in insulin secretion of oral *versus* intravenous glucose injection (39). The dose-dependent effect of incretins on the insulin secretion rate is also diminished in T2D patients (40). Our GIRKO HFD mouse model recapitulated this pathology (*i.e.*, reduced glucose-lowering effect of exendin-4) and suggests that impairments to the incretin effect could be an additional factor promoting the progression of diabetes. Thirdly, our results suggested HFD increased inflammatory molecules (LPS) in circulation. Whether the GIRKO mice responded to this and restricted adipose tissue expansion with increased inflammation and adipocyte apoptosis warrants future investigations. Lastly, we showed diet had profound impact on gut microbiome composition in Control and GIRKO mice. HFD decreased the relative abundance of *Bacteroidetes* and increased the relative abundance of *Firmicutes* in both groups. Studies reported that HFD significantly elevated the proportions of the phylum *Actinobacteria* and the class *Actinobacteria_c* in a positive association with weight gain and obesity in animals and humans (41, 42). However, our results did not support this. We showed that GIRKO mice on HFD had increased *Actinobacteria* but less body weight. Prior to HFD, GIRKO and control mice had similar body weights. After HFD, Control mice gained the most weight, while GIRKO mice gained less. Despite these trends in body weight change, GIRKO mice on HFD had the greatest abundance of *Actinobacteria*, suggesting that these were likely the result of the interaction between diet and genotype. Mucin-degrading *Actinobacteria* and its abundance might be associated with reduced gut barrier function on HFD, which in turn contributes to the pathophysiology of diabetes (30). Therefore, the increased abundance of *Actinobacteria* in the gut microbiome of GIRKO mice on HFD is interesting and warrants future investigation.

Tissue-specific models of Insr deletion have been used to make important discoveries regarding the complex nature of insulin signaling for regulating glucose homeostasis. For example, Muscle-Insr-KnockOut (MIRKO) mice have seemingly normal peripheral insulin sensitivity and glucose tolerance (15), despite having reduced insulin kinase activity. Follow-up studies in MIRKO mice found that glucose uptake in response to exercise was also normal, and that synergistic effects of insulin plus exercise were also normal (43). Together, these studies support the notion that Insr-independent mechanisms were responsible for glucose uptake in these mice.

FIRKO (fat-specific insulin receptor knockout) mice were previously generated to disrupt Insr expression in adipocytes (16). In general, FIRKO mice appeared to have an improved metabolic phenotype, were protected from hyperphagic obesity, had decreased adiposity, improved i.p. glucose tolerance, and altered leptin expression. Although GIRKO mice also have Insr-knockout in adipocytes, a key difference is that we observed impaired glucose tolerance in the context of high dietary fat intake. On chow diet, FIRKO mice had lower fat mass and were better protected from age-related and brain lesion-induced obesity and glucose intolerance. GIRKO mice gained less weight and fat mass on HFD compared with Control mice. Insulin is an important anabolic hormone to promote glucose uptake, lipogenesis, glycogenesis, and protein synthesis in insulin sensitive tissues, including adipose tissue and muscle. Therefore, we reason that the major cause for decreased weight of GIRKO mice on HFD was a result of the loss of insulin signaling. The impact of loss of Insr on adipocyte size was observed in both FIRKO and GIRKO mice. The adipose tissue of FIRKO mice exhibited polarization of adipocytes into populations of large and small cells, while the adipose tissue of GIRKO mice was also more heterogeneous with relatively less small adipocytes and more large adipocytes. Consistent with FIRKO mice, no difference in the *Pparg* expression was observed in GIRKO fat tissue. In contrast to the decreased Srebp-1, Cebpa, and Fas protein expression in FIRKO fat tissue, the transcripts of *Srebp1c*, *Acc*, *Plin1*, and *Hsl* were increased in the GIRKO adipose tissue. Therefore, we conclude that the reason of smaller adipose tissue in GIRKO compared with control mice was not due to the defective expression of genes controlling adipocyte differentiation. The increased adipogenic and lipid handling gene expression likely reflected a compensatory feedback mechanism in transcriptional responses. During our study, we measured the leptin/fat mass relationship in HFD-fed GIRKO mice but determined that the correlation between these two variables was not changed. Based on the differences in leptin/fat mass relationship between the two models, secretion of endocrine factors such as leptin may depend on the overall metabolic phenotype of the mouse model rather than Insr signaling itself in adipocytes. Finally, despite reduced adiposity, GIRKO mice on HFD had impaired oral glucose tolerance, fatty liver disease, and insulin resistance in other tissues, which were collectively different from the previous reported models.

Using GIRKO mice as a model of insulin resistance, we identified additional factors/mechanisms responsible for adverse metabolic effects that drive the ultimate progression of frank diabetes. The overt hyperglycemia phenotype in HFD-fed GIRKO mice was a result of the impaired hierarchical regulatory mechanisms maintaining glucose homeostasis. First, knocking out insulin receptor in Glut4-expressing tissues conferred insulin resistance and predisposed mice to defective glucose metabolic homeostasis. Secondly, hyperinsulinemia caused by systemic insulin resistance led to the compensation and stress of pancreatic islets, which eventually manifested as blunted incretin response. Thirdly, dyslipidemia shown as reduced fat mass, ectopic hepatic lipid deposition and secretion contributes to lipotoxicity in organs with key metabolic function. Lastly, inflammatory cues generated by the gut, which was the first organ encountering HFD, may permeate to other tissues by circulation and further exacerbate the metabolic defects. Collectively, our combined genetic and HFD model illustrates a potential diet–genotype interaction that improves our understanding of the underpinning between diets and diabetes pathophysiology at the organismal level and thus helps to identify molecular targets for developing effective therapeutic strategies.

## Experimental procedures

### Experimental animals

Mice were maintained on a 12:12 h light:dark cycle in the Lab Animal Resource Center (LARC) facility at Indiana University School of Medicine. GIRKO mice were generated on a mixed background derived from 129/Sv, C57BL/6, and FVB as previously described (17). Cre negative littermates were used as controls. GIRKO and Control mice were hemizygous for *Insr* (*i.e.*, *Insr*^*lox/Δ*^). Mice were euthanized with CO_2_ and tissues were stored at −80 °C. All animal procedures were followed and approved by Indiana University School of Medicine Animal Care and Use Committee (IACUC #11121, 19013).

### Genotyping

Mouse genotyping of the *Insr* allele was performed using PCR primers that detect wild-type (*+*), flanking *loxP* sites (*lox*), and recombined (*Δ*) alleles. Primer names and sequences were as follows: P5 5′-CGCCTACACATCACATGC-3′; P8 5′-TCCACATTTTACCAACCCTGTCAC-3′; P10 5′-CCTGGTATAAGTCTCTCATTTGG-3′. Temperature cycles were performed as follows: 94 °C (3 min), then 34 cycles of 94 °C (30 s), 55 °C (30 s), 72 °C (30 s), followed by 72 °C (5 min), followed by electrophoresis (2% agarose/TBE gel). Band sizes for *+*, *lox*, and *Δ* alleles were 100 bp, 250 bp, and 400 bp, respectively. Genotyping of the *GLUT4-Cre* transgene was performed as previously described (17).

### Dietary treatment

Prior to HFD exposure, experimental animals were raised on 62.1% of calories from carbohydrates, 24.6% from protein, and 13.2% from fat (LabDiet #5053). At 7 months old, mice started HFD containing 60% calories from fat, 20% from protein, 20% from carbohydrate (Research Diets, D12492). Mice were fed *ad libitum* unless noted otherwise for short fasting (5–6 h), overnight fasting (16 h), or refeeding studies. EchoMRI-500 was used for live animal body scans to determine fat and lean mass.

### Glucose measurements

Blood glucose was sampled from the tail vein and measured with an AlphaTRAK 2 glucometer. Glucose tolerance tests were performed by oral gavage (OGTT) or intraperitoneal injection (IPGTT). Mice were fasted for 5–6 h during the day for these experiments with one exception for overnight fasting (∼16 h) in NCD-fed mice during OGTT. For experiments using exendin-4 pretreatment, exendin-4 (6 μg/kg) was injected 20 min before the intraperitoneal glucose tolerance test (IPGTT). One week before, the same procedure was used for IPGTT with vehicle pretreatments.

### Serum biochemistries

Blood was collected from tail or heart during *ad libitum* feeding or as indicated by the figure legend. Leptin was measured from serum collected at 12 weeks under *ad libitum* feeding condition. Serum insulin during *ad libitum* feeding was measured immediately prior to starting HFD and at 4 and 12 weeks of HFD feeding. Serum insulin and leptin were measured using ELISA (EMD Millipore). Serum triglycerides were measured *via* Serum TG assay (Thermo Fisher). Total GLP-1 in serum was measured by immunoassay (Meso Scale Discovery). All reactions were performed according to manufacturer protocols.

### Immunohistochemistry

Pancreata were fixed by cardiac perfusion with 4% paraformaldehyde; adipose and livers were fixed in 4% paraformaldehyde without cardiac perfusion. Formalin-fixed paraffin-embedded (FFPE) sections stained with hematoxylin and eosin were prepared by the Indiana University School of Medicine Histology Core using standard protocols; sections were cut at 5-μm thickness.

### Islet area quantification

FFPE pancreatic sections deparaffinized with xylenes and rehydrated with decreasing concentrations of EtOH (100%, 95%, 90%, 80%, 70%) and water, then incubated in anti-insulin primary antibodies (Abcam ab181547, 1:500) overnight at 4 °C. Insulin was labeled with anti-rabbit ImmPRESS reagent and NovaRed substrate kit. Hematoxylin was used to counterstain tissue. Data shown are an average of two sections per pancreas, separated by at least 60 μm.

### (GSIS) in *ex vivo* islets

Mouse pancreatic islets were isolated by collagenase digestion as previously described (44) from 15-week-old female GIRKO mice given HFD for 4 weeks. After isolation, islets were allowed to recover overnight. Fifty handpicked islets per mouse were loaded into the Biorep Perifusion System with each chamber containing islets belonging to unique individuals. Islets were perifused at a rate of 120 μl/min with Krebs buffer containing 2.8 mmol/L glucose for 20 min, 16.7 mmol/L glucose for 30 min, followed by washout for 6 min, and chased for 20 min with 30 mM potassium chloride as a depolarizing stimulus. Recovered islets were lysed by shearing with a 26 1/2G sterile needle in lysis buffer containing 1% NP-40, 0.05% deoxycholate, 0.1% SDS, 0.2% Sarkosyl, 10% Glycerol, 1 mM DTT, 1 mM EDTA, 10 mM NaF, 50 mM Tris (pH 8.0), 1× Complete protease inhibitor cocktail (Roche), and 1× PhosStop inhibitor cocktail (Roche) in PBS. Secreted insulin was measured using ELISA (Mercodia), and results were normalized to total insulin content in islet lysates.

### Microscopy

For imaging and analysis an AxioScan.Z1 with 5× objective was used to acquire digital images of the entire stained longitudinal pancreatic section. β-cell area percentage was quantified using Zen 2 (Zeiss) software by measuring the insulin positive area (in pixels) and pancreas area (in pixels). Representative images of adipose and hepatic tissues were collected at 5× and 20× magnification on a Leica DMi1 light microscope.

### Adipocyte morphology

Images were collected using an AxioScan.Z1 at 40× magnification and saved at 0.878 μm^2^ per pixel resolution. Image analysis was performed using FIJI (ImageJ) software with MorphoLibJ integrated library and plugin. Quantification of hematoxylin and eosin-stained sections was achieved using a multistep protocol. First, image preprocessing steps were to convert RGB color images to 8 bit grayscale, followed by background subtraction using the run(“Subtract Background…”, “rolling = 20 sliding”) command. Segmentation was performed in MorphoLibJ using the Morphological Segmentation tool with the following settings: type = “object”, radius = 3, GradientType = “Morphological”, tolerance = 4.0, calculateDams = true, connectivity = 4, DisplayFormat = “Overlaid dams.” Segmented images were smoothed using the run (“Gaussian Blur…”, “sigma = 2”) command and converted to binary images with upper and lower thresholds at 255 and 190, respectively. Regions of interest (ROIs) were registered using the run (“Analyze Particles…”, “size = 100–2000 display exclude clear add”) command. False-positive ROIs along the edges were deleted prior to analysis. The area of each ROI was measured and exported as an Excel table for analysis.

### Indirect calorimetry

Indirect calorimetry measurements were collected using a TSE PhenoMaster Platform as described previously (45). Briefly, mice were single-housed for a 48-h acclimation period before data were recorded for analysis. Metabolic parameters (food intake, energy expenditure, respiratory exchange ratio) were measured at 51 min intervals during a normal 12-h light:dark cycle. Total body weight and lean mass were determined beforehand with an echoMRI-100 for calculations.

### Hepatic triglycerides and glycogen assays

Hepatic triglycerides were extracted from liver homogenates using the Folch method (46) and measured with a TG colorimetric assay kit. Hepatic glycogen was measured from new liver homogenate preparations following amyloglucosidase digestion with a Glucose Assay Kit (Sigma-Aldrich).

### Hepatic very-low-density lipoprotein (VLDL) secretion assay

Tyloxapol (Triton WR1339) was dissolved in isotonic saline 10% (V/V) before use. Mice (8 males and 14 females) were fasted for 4 h. Mice body weight was measured, and the baseline arterial blood samples were drawn. Tyloxapol (400 mg/kg) was injected into the tail vein of mice. The blood samples were collected 1 h or 2 h after tyloxapol injection, and serum triglyceride was measured immediately.

### mRNA quantitation

The transcription of *Pparg, Cebpa, Srebp1c, Acc, Plin1, Hsl, Fabp4, Lep, Adipoq, G6pc, Fbpase, Gck1, Pck1, Pklr, Pdk4, Scd1, Fas, Chrebp* was quantified by reverse transcription and quantitative real-time PCR (RT-PCR). RNA was extracted with TRIzol Reagent. Superscript II reverse transcriptase was used to synthesize template cDNA. Gene-specific primers used during RT-PCR spanning introns were validated by melting curve analysis and gel electrophoresis. Primer sequences are available upon request. All reactions were performed according to manufacturer protocols.

### RNA-sequencing library preparation and sequencing

RNA was extracted from duodenum tissue with Qiagen RNeasy Plus Mini Kit and evaluated for quantity and quality for a minimum RIN score of 7 or higher using Agilent Bioanalyzer 2100. Two-hundred nanograms of total RNA per sample was used for library preparation. cDNA libraries were prepared using RNA fragmentation, cDNA synthesis, ligation of index adaptors, and amplification using KAPA mRNA Hyperprep Kit (KK8581). Each library was quantified, and its quality accessed by Qubit and Agilent 2100 Bioanalyzer. Total RNA was sequenced with the 2 × 75 paired-end configuration on an Illumina HiSeq 4000 with an average of 30.6 M reads. A Phred quality score (Q score) was used to measure the quality of sequencing. More than 90% of the sequencing reads reached Q30 (99.9% base call accuracy).

### RNA-sequencing alignment and analysis

All sequenced libraries were then mapped to the mouse genome (UCSC mm10) using STAR RNA-seq aligner (47). The reads distribution across the genome was assessed using bamutils (from ngsutils) (48). Uniquely mapped sequencing reads were assigned to mm10 refGene genes using featureCounts (49). The sequencing data were deposited in IUPUI DataWorks (50). Differential expression (DE) analyses were performed using edgeR v3.22.3 implemented in the Bioconductor package (51) to identify differentially expressed mRNAs between Control and GIRKO samples. Biological coefficients of variation between the samples were estimated using an empirical Bayes approach under the assumption that the data follows a negative binomial distribution. Low expression transcripts were filtered out based on percentage of samples (less than 50%) and CPM cutoff of 0.5. Statistical significance was defined as *p*-value ≤ 0.05 and filtered by fold change (FC) ≥ 2 of expression between GIRKO and Control mice. Pathway analysis was conducted in Ingenuity Pathway Analysis Software (Qiagen).

### Micro-computed tomography (micro-CT) of femurs

After euthanasia at 44 weeks of age, the right femur was dissected from each mouse, fixed for 2 days in 10% neutral buffered formalin, and then transferred into 70% ethanol for micro-CT scanning on a high-throughput micro-CT specimen scanner (micro-CT-35; Scanco Medical AG). The middle 15% and distal 33% of each femur were scanned using the following conditions: 50 kV, 120 mA, 151-ms integration time, and 10-μm voxel resolution. Three-dimensional morphometric properties of the cancellous bone in the distal femur were measured as previously described (52). Analyses of cortical bone parameters were collected using a 20-slice stack that was centered on the midshaft slice of the femur, as previously described (52).

### 16S rRNA sequencing of gut microbes

Fecal samples were collected from unique individuals before and after 4 weeks of HFD treatment and sent to University of Missouri DNA Core Facility for library preparation and sequencing. Detailed protocols describing library preparation, sequencing, and informatics were described previously (53). Briefly, the V4 region of bacterial 16S rRNA gene was used to generate amplicon libraries with dual-indexed primers (U515 F/806R), flanked by Illumina standard adapter sequences (54, 55). Amplification and cleanup were carried out as previously described (53). Final amplicon pools were evaluated using an Advanced Analytical Fragment Analyzer automated electrophoresis system, quantified using a Qubit 2.0 fluorometer and quant-iT HS dsDNA kits, and diluted according to Illumina's standard protocol for sequencing on the MiSeq instrument using V2 chemistry kits. Amplicon sequence variants (ASVs) were assembled and annotated as previously described (53), using the Qiime2 dada2 plugin (version 1.10.0) to denoise, dereplicate, and count amplicon sequence variants (ASVs) (56). R version 3.5.1 and Biom version 2.1.7 were used in Qiime2. Taxonomies were assigned using the Silva.v132 database (57), using the classify-sklearn procedure. The results were deposited and accessible in NCBI Sequence Read Archive (SRA) (Submission ID SUB9616899, BioProject ID PRJNA729467).

### Statistical analysis of amplicon sequence variants

Statistical analyses of beta-diversity in microbiomes were performed as previously described (53) using Past 3.26 b. In brief, the relative ASV abundances were ¼ root-transformed for statistical comparisons and compared using one-way permutational multivariate analysis of variance (PERMANOVA) of Bray–Curtis similarities. Pairwise statistics were calculated and *p*-values adjusted using Bonferroni's method.

### Equipment, reagents, and software

Detailed information for products, equipment, and software can be found in supporting information.

### Statistical comparisons

Data shown are mean ± SEM. Detailed information regarding the statistics for each figure can be found in the legends and in supporting information.

## Data availability

All data are contained within the manuscript.

## Supporting information

This article contains supporting information.

## Conflict of interest

The authors declare that they have no conflicts of interest with the contents of this article.
