# Supplementary material for: A high-fat diet catalyzes progression to hyperglycemia in mice with selective impairment of insulin action in Glut4-expressing tissues
Source: J Biol Chem. 2021 Nov 18;298(1):101431. doi: 10.1016/j.jbc.2021.101431 (PMC8689209; doi:10.1016/j.jbc.2021.101431)
Supplement: Figures S1–S5 and Table S1 [file mmc1.pdf]

**Supporting information**

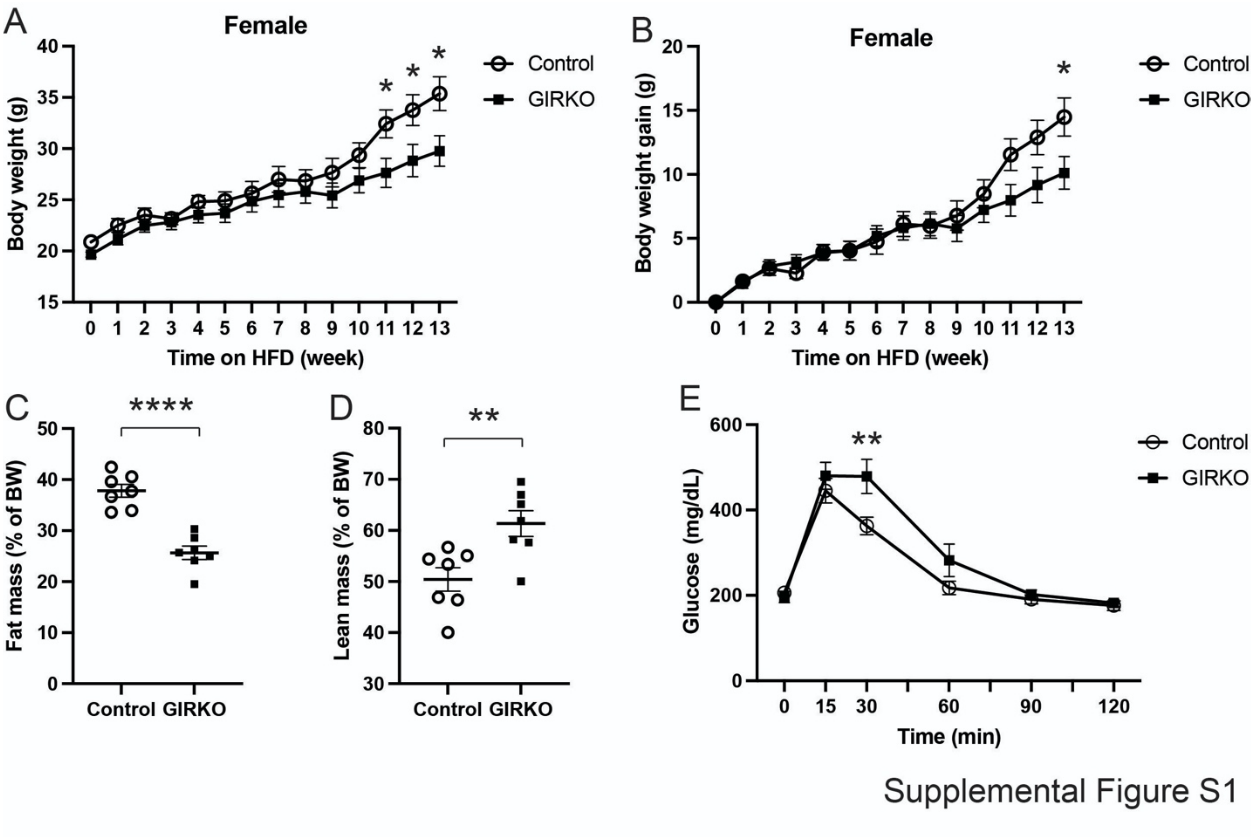


Figure S1. Female GIRKO mice had lower body weight, gained less adiposity, and reduced glucose tolerance after longer-term HFD feeding.

A) Weekly body weight in female mice measured from the beginning of HFD administration.

B) Weekly change in body weight in female mice since beginning HFD.

C) Lean mass percentage of total body weight (BW).

D) Fat mass percentage of total body weight (BW).

E) Oral glucose tolerance test (OGTT, 3.3 g per kg lean body mass) in mice given HFD.

Data shown are Mean ± SEM. Statistical comparisons were performed in panels A-D using Student's t-test and panel E using 2way ANOVA. (*) indicates $p<0.05$, (**) indicates $p<0.01$, (****) indicates $p<0.0001$,  n = 7 Control, 7 GIRKO for all panels.

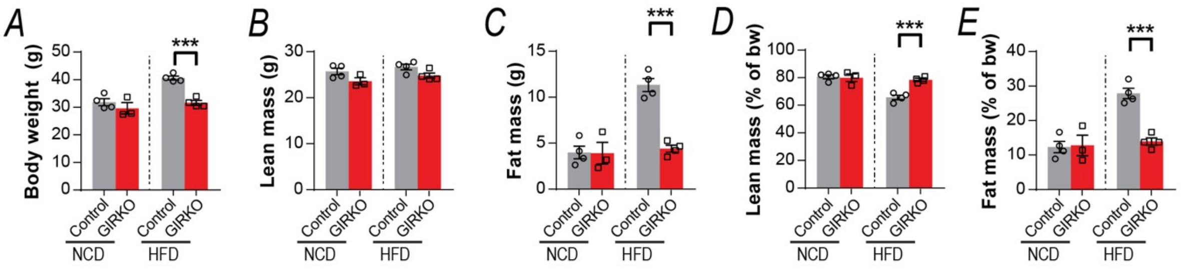


Supplemental Figure S2

Figure S2. Body weight and composition analysis of age-matched male cohorts. Age-matched male cohorts were randomly assigned to NCD or HFD for body composition analysis.

A) Total body weight in grams.

B) Lean body mass in grams.

C) Fat mass in grams.

D) Lean mass percentage of total body weight (bw).

E) Fat mass percentage of total body weight (bw).

Data shown are Mean ± SEM. Statistical comparisons in panels A-B were performed using Student's t-test was used for panels C-D. (***) indicates $p<0.001$, n = 8 Control, 9 GIRKO for all panels.

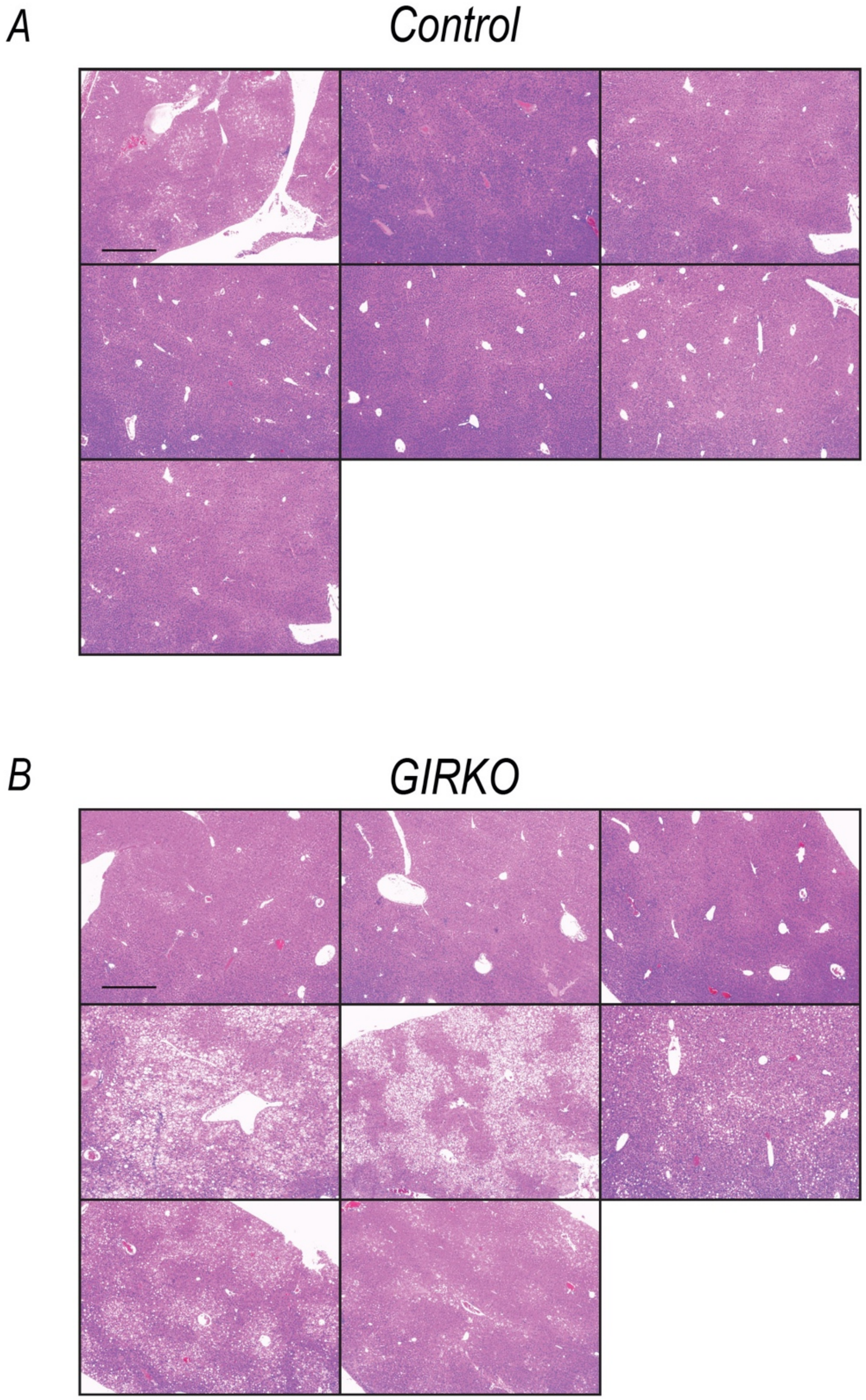


Supplemental Figure S3

Figure S3. Highly penetrant hepatic steatosis in GIRKO mice fed high-fat diet.

Hematoxylin and eosin-stained hepatic tissue sections. Each panel shows a different liver that is from Control (A) or GIRKO (B) animals. Scale bar is 500 µm.

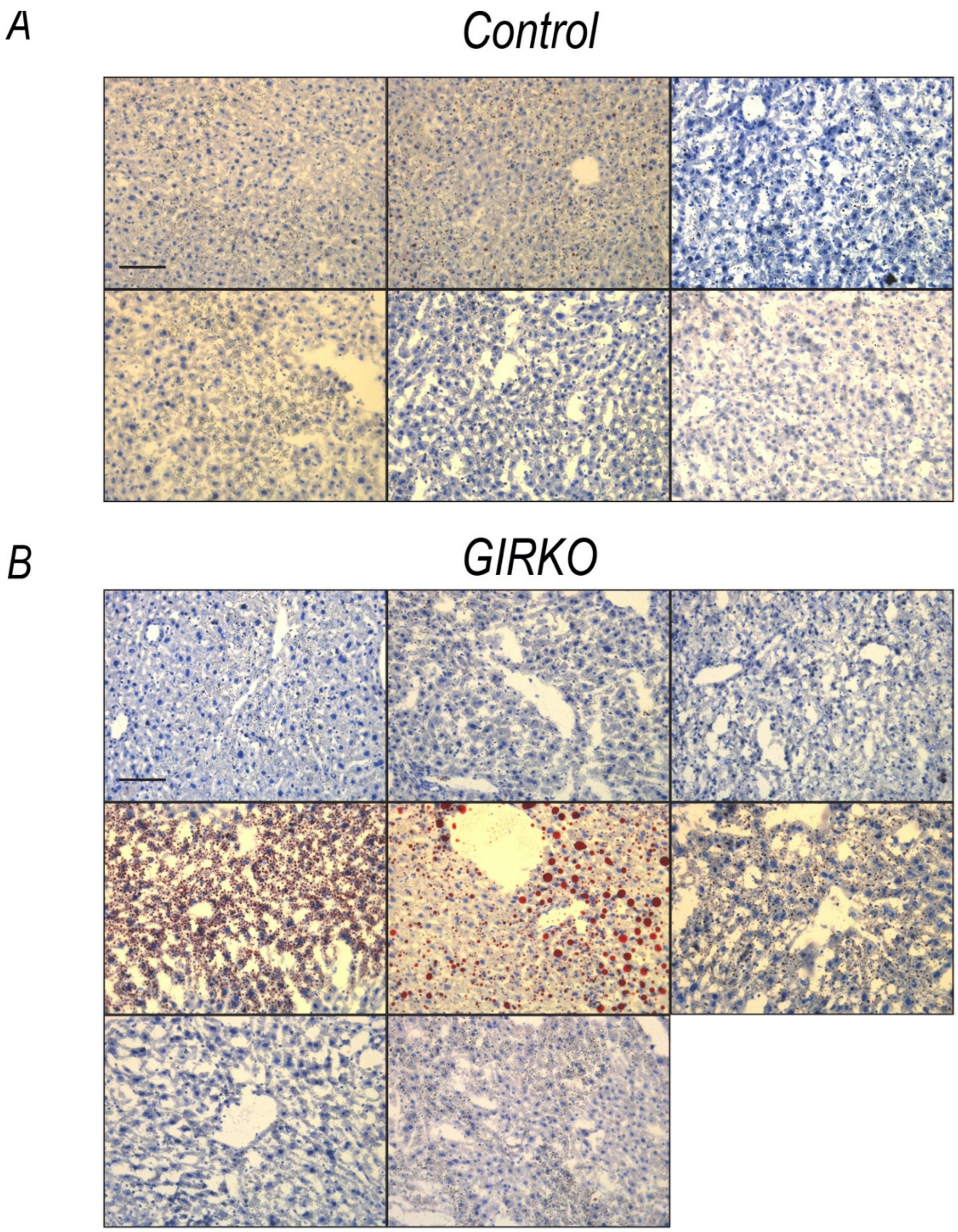


Supplemental Figure S4

Figure S4. Oil Red O stain of hepatic tissue sections.
Oil red O-stained hepatic tissue sections. Each panel shows a different liver that is from Control (A) or GIRKO (B) animals. Scale bar is 100 μm.

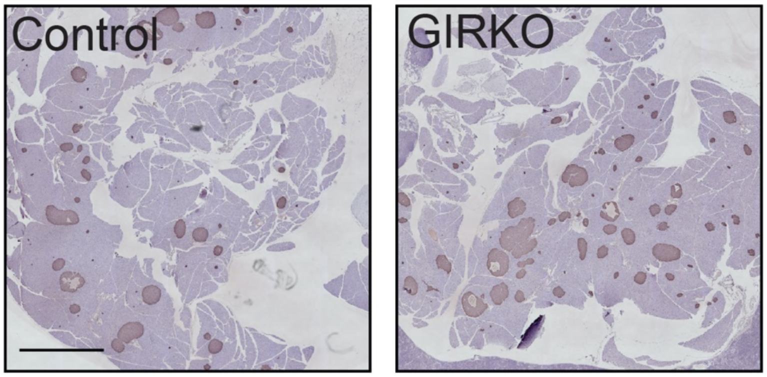


Supplemental Figure S5

Figure S5. Representative images of pancreatic tissue sections.

Representative immunohistological staining of pancreatic tissues from Control or GIRKO animals. Insulin-positive β-cells were stained dark purple by immunohistochemistry and counterstained with hematoxylin and eosin stain. Scale bar is 2 mm.

| **High-fat Diet, 14 weeks** | | **Control (n=8)** | | **GIRKO (n=9)** | | |
|---|---|---|---|---|---|---|
| | | **average** | **SEM** | **average** | **SEM** | ***p*** |
| **Distal Femur** | | | | | | |
| **BV/TV (%)** | **bone volume fraction** | 7.31 | 0.61 | 9.47 | 1.07 | - |
| **BMD (mg/cm$^3$)** | **density of bone pixels** | 929.38 | 7.19 | 924.35 | 5.33 | - |
| **Tb.Th (mm)** | **thickness of struts** | 0.05 | 0.00 | 0.05 | 0.00 | - |
| **BMC (mg)** | **bone mineral content** | 0.27 | 0.03 | 0.34 | 0.04 | - |
| **Tb.N (1/mm)** | **number of struts** | 2.90 | 0.11 | 3.24 | 0.09 | 0.04 |
| **Tb.Sp (mm)** | **distance between struts** | 0.34 | 0.01 | 0.30 | 0.01 | 0.03 |
| **Femur Midshaft** | | | | | | |
| **BMD (mg/cm$^3$)** | **density of bone pixels** | 1212.56 | 5.14 | 1218.68 | 5.40 | - |
| **Cort.Th** | **thickness** | 0.21 | 0.00 | 0.21 | 0.00 | - |
| **Imin (mm$^4$)** | **minimum moment of inertia** | 0.22 | 0.01 | 0.23 | 0.01 | - |
| **Imax (mm$^4$)** | **maximum moment of inertia** | 0.42 | 0.02 | 0.42 | 0.02 | - |
| **pMOI (mm$^4$)** | **polar moment of inertia** | 0.64 | 0.03 | 0.66 | 0.02 | - |
| **TArea (mm$^2$)** | **total area** | 2.34 | 0.06 | 2.37 | 0.04 | - |
| **Barea (mm$^2$)** | **bone area** | 1.07 | 0.02 | 1.09 | 0.03 | - |

Table S1. Effects of 3 months of high-fat diet on bone morphometry. Statistical comparisons were performed using student's t-test.
